# Checklist of the Diptera superfamilies Tephritoidea and Sciomyzoidea of Finland (Insecta)

**DOI:** 10.3897/zookeys.441.7143

**Published:** 2014-09-19

**Authors:** Jere Kahanpää, Kaj Winqvist

**Affiliations:** 1 Finnish Museum of Natural History, Zoology Unit, P.O. Box 17, FI–00014 University of Helsinki, Finland; 2 Mikonkatu 3 C 52, FI–20100 Turku, Finland

**Keywords:** Finland, Diptera, checklist, Tephritoidea, Sciomyzoidea

## Abstract

A revised checklist of the flies of superfamilies Tephritoidea and Sciomyzoidea of Finland is provided. The following families are covered: Eurygnathomyiidae, Lonchaeidae, Neottiophilidae, Pallopteridae, Piophilidae, Platystomatidae, Tephritidae, Ulidiidae (Tephritoidea); Coelopidae, Dryomyzidae, Heterocheilidae, Phaeomyiidae, Sciomyzidae, Sepsidae (Sciomyzoidea).

## Introduction

With over 7800 known extant species (Pape et al. 2011), Tephritoidea is one of the larger Diptera superfamilies. The nominotypical family of Tephritoidea, the fruit flies (Tephritidae), includes over half of the currently known species in the superfamily. The highest diversity of tephritoids occurs in the tropics. Six of the nine extant tephritoid families have been found in Finland: no richardiid, ctenostylid or pyrgotid have been found in the country. The Tephritidae, Ulidiidae and Platystomatidae (plus Pyrgotidae) form a well-defined, probably monophyletic group. The other tephritoid families are more basal in their phylogenetic position (Korneyev 1999). The exact relationships between the families are far from settled and some authors exclude Pallopteridae and Eurygnathomyiidae from the superfamily (Wiegmann et al. 2011). Several subfamily schemes have been proposed for Tephritidae. The classification by Korneyev (2000) is adopted here. For Pallopteridae, Piophilidae (with Neottiophilidae) and Ulidiidae we follow McAlpine (1981), Ozerov (2005) and Kameneva and Korneyev (2006) respectively. Norrbom et al. (1999) provide a catalogue of world tephritid names. The Finnish tephritoid flies were last listed by Winqvist and Kahanpää (2007). Hackman (1956) reviewed the Lonchaeidae of Eastern Fennoscandia. This paper is now somewhat outdated, but still valuable.

Sciomyzoidea is a smaller superfamily with some 1150 described species (Pape et al. 2011). It includes two medium-sized families (Sciomyzidae and Sepsidae) and nine smaller families comprising at most 30 species. The millipede parasitoids of family Phaeomyiidae are sometimes classified as a subfamily of Sciomyzidae. The coelopids and heterocheilids, with just one species each in Finland, are closely associated with stranded marine seaweed. The family placement of *Heterocheila* has been rather unstable: during the last 40 years it has been placed in Helcomyzidae, Dryomyzidae and Coelopidae or regarded as having a separate family status. Here we follow the recent world catalogue (Mathis 2011) and keep Heterocheilidae as a separate family. The family name Heterocheilidae is preoccupied by Heterocheilidae Railliet & Henry, 1915 in Nematoda (Mathis 2011). This homonymy has not yet been resolved.

World catalogues have recently been published for Coelopidae (Mathis and McAlpine 2011) Dryomyzidae (Mathis and Sueyoshi 2011), Heterocheilidae (Mathis 2011), Sciomyzidae (Rozkošný 1995) and Sepsidae (Ozerov 2005). The Finnish sciomyzoid flies were last listed by Winqvist and Kahanpää (2007).

**Table 1.** Number of species in tephritoid and sciomyzoid families.

Family	Number of species in			Level of knowledge
	World (Pape et al. 2011)	Europe (Pape and Beuk 2013)	Finland	
**Tephridoidea:**				
Eurygnathomyiidae	1	1	1	good
Lonchaeidae	504	96	41–44	average
Neottiophilidae	4	2	2	average
Pallopteridae	70	22	13	good
Piophilidae	78	27	15	average
Platystomatidae	1162	21	2	good
Tephritidae	4712	267	69	average–good
Ulidiidae	675	106	16	good
**Sciomyzoidea:**				
Coelopidae	35	3	1	average
Dryomyzidae	22	5	5	average
Heterocheilidae	2	1	1	average
Phaeomyiidae	3	3	2	good
Sciomyzidae	605	136	73–74	good
Sepsidae	340	48	32	good

## Checklist part 1: Tephritoidea

suborder Brachycera Macquart, 1834

clade Eremoneura Lameere, 1906

clade Cyclorrhapha Brauer, 1863

infraorder Schizophora Becher, 1882

clade Muscaria Enderlein, 1936

parvorder Acalyptratae Macquart, 1835

superfamily Tephritoidea Newman, 1834

**EURYGNATHOMYIIDAE** Czerny, 1934

***EURYGNATHOMYIA*** Czerny, 1904

*Eurygnathomyia bicolor* (Zetterstedt, 1837)

**LONCHAEIDAE** Rondani, 1856

DASIOPINAE Morge, 1963

Dasiopini Morge, 1963

***DASIOPS*** Rondani, 1856

*Dasiops appendiculus* Morge, 1959

*Dasiops facialis* Collin, 1953

*Dasiops mucronatus* Morge, 1959

= *hennigi* misid.

= *latiterebra* misid.

*Dasiops occultus* Collin, 1953

= *albiceps* (Frey, 1930) preocc.

*Dasiops perpropinquus* Morge, 1959

*Dasiops spatiosus* (Becker, 1895)

*Dasiops trichosternalis* Morge, 1959

EAROMYIINAE Morge, 1963

***CHAETOLONCHAEA*** Czerny, 1934

*Chaetolonchaea pallipennis* (Zetterstedt, 1855)

= *dasyops* misid.

***EAROMYIA*** Zetterstedt, 1842

*Earomyia lonchaeoides* Zetterstedt, 1848

*Earomyia schistopyga* Collin, 1953

*Earomyia viridana* (Meigen, 1826)

***PROTEAROMYIA*** McAlpine, 1962

*Protearomyia nigra* (Meigen, 1826)

LONCHAEINAE Rondani, 1856

Lonchaeini Rondani, 1856

***LONCHAEA*** Fallén, 1820

*Lonchaea affinis* Malloch, 1920

= *laxa* auct. nec Collin, 1953

*Lonchaea albigena* Collin, 1953

*Lonchaea albitarsis* Zetterstedt, 1838

? *Lonchaea bruggeri* Morge, 1967

*Lonchaea bukowskii* Czerny, 1934

*Lonchaea caledonica* MacGowan & Rotheray, 2000

= *laticornis* auct. nec Meigen, 1826

*Lonchaea carpathica* Kovalev, 1974

*Lonchaea chorea* (Fabricius, 1781)

*Lonchaea collini* Hackman, 1956

? *Lonchaea contigua* Collin, 1953

*Lonchaea corusca* Czerny, 1934

= *alni* Ringdahl, 1947

= *lauta* Collin, 1953

= *britteni* Collin, 1953

*Lonchaea defecta* McAlpine, 1964

*Lonchaea deutschi* Zetterstedt, 1838

= *sarekensis* Frey, 1916

? *Lonchaea difficilis* Hackman, 1956

*Lonchaea fraxina* MacGowan & Rotheray, 2000

*Lonchaea freyi* Czerny, 1934

*Lonchaea fugax* Becker, 1895

= *cariecola* Czerny, 1934

*Lonchaea hackmani* Kovalev, 1981

= *peregrina* auct. nec Becker, 1895

*Lonchaea ipsiphaga* McAlpine, 1964

= *maniola* misid.

*Lonchaea limatula* Collin, 1953

= *flavidipennis* auct. nec Zetterstedt, 1847

*Lonchaea nitens* (Bigot, 1885)

= *krogerusi* Czerny, 1934

*Lonchaea palposa* Zetterstedt, 1847

*Lonchaea patens* Collin, 1953

*Lonchaea ragnari* Hackman, 1956

*Lonchaea scutellaris* Rondani, 1874

*Lonchaea sororcula* Hackman, 1956

*Lonchaea stackelbergi* Czerny, 1934

*Lonchaea subneatosa* Kovalenko, 1974

*Lonchaea sylvatica* Beling, 1873

= *lucidiventris* Becker, 1895

*Lonchaea tarsata* Fallen, 1820

*Lonchaea ultima* Collin, 1953

*Lonchaea zetterstedti* Becker, 1902

**NEOTTIOPHILIDAE** Hendel, 1916

***ACTENOPTERA*** Czerny, 1904

*Actenoptera hilarella* (Zetterstedt, 1847)

***NEOTTIOPHILUM*** Frauenfeld, 1868

*Neottiophilum praeustum* (Meigen, 1826)

**PALLOPTERIDAE** Loew, 1862

***PALLOPTERA*** Fallén, 1820

*Palloptera anderssoni* Rotheray & MacGowan, 1999

*Palloptera formosa* Frey, 1930

*Palloptera marginata* (Meigen, 1826)

= *costalis* Loew, 1873

*Palloptera umbellatarum* (Fabricius, 1775)

= *arcuata* auct. nec (Fabricius, 1781)

*Palloptera ustulata* Fallén, 1820

***TEMNOSIRA*** Enderlein, 1936

*Temnosira ambusta* (Meigen, 1826)

*Temnosira saltuum* (Linnaeus, 1758)

***TOXONEURA*** Macquart, 1835

*Toxoneura ephippium* (Zetterstedt, 1860)

*Toxoneura laetabilis* (Loew, 1873)

*Toxoneura modesta* (Meigen, 1830)

= *umbellatarum* auct. nec (Fabricius, 1775)

*Toxoneura trimacula* (Meigen, 1826)

*Toxoneura usta* (Meigen, 1826)

*Toxoneura venusta* (Loew, 1858)

= *atriventris* (Ringdahl, 1947)

**PIOPHILIDAE Macquart, 1835**

PIOPHILINAE Macquart, 1835

***ALLOPIOPHILA*** Hendel, 1917

= ***Arctopiophila*** Duda, 1924

= ***Boreopiophila*** Frey, 1930

= ***Parapiophila*** McAlpine, 1977

*Allopiophila calceata* (Duda, 1924)

*Allopiophila flavipes* (Zetterstedt, 1847)

*Allopiophila lonchaeoides* (Zetterstedt, 1838)

*Allopiophila luteata* (Haliday, 1833)

*Allopiophila pectiniventris* (Duda, 1924)

*Allopiophila tomentosa* Frey, 1930

*Allopiophila vulgaris* (Fallén, 1820)

*Allopiophila* sp. A

***AMPHIPOGON*** Wahlberg, 1845

*Amphipogon flavus* (Zetterstedt, 1838)

= *spectrum* Wahlberg, 1845

***MYCETAULUS*** Loew, 1845

*Mycetaulus bipunctatus* (Fallén, 1823)

***PIOPHILA*** Fallén, 1810

*Piophila casei* (Linnaeus, 1758)

***PROCHYLIZA*** Walker, 1849

= ***Liopiophila*** Duda, 1924

*Prochyliza nigrimana* (Meigen, 1826)

*Prochyliza varipes* (Meigen, 1830)

= *lundbecki* misid.

***PSEUDOSEPS*** Becker, 1902

*Pseudoseps signata* (Fallén, 1820)

***STEARIBIA*** Lioy, 1864

*Stearibia nigriceps* (Meigen, 1826)

= *foveolata* (Meigen, 1826)

= *coerulescens* (Zetterstedt, 1847)

**PLATYSTOMATIDAE** Schiner, 1862

***PLATYSTOMA*** Meigen, 1803

*Platystoma seminationis* (Fabricius, 1775)

***RIVELLIA*** Robineau-Desvoidy, 1830

*Rivellia syngenesiae* (Fabricius, 1781)

**TEPHRITIDAE** Newman, 1834

TRYPETINAE Loew, 1861 *sensu lato*

Adramini Hendel, 1914

***EUPHRANTA*** Loew, 1862

**sg. *Euphranta*** Loew, 1862

*Euphranta connexa* (Fabricius, 1794)

**sg. *Rhacochlaena*** Loew, 1862

*Euphranta toxoneura* (Loew, 1846)

Trypetini Loew, 1861

***ACIDIA*** Robineau-Desvoidy, 1830

*Acidia cognata* (Wiedemann, 1817)

***ANOMOIA*** Walker, 1835

= ***Phagocarpus*** Rondani, 1870

*Anomoia purmunda* (Harris, 1776)

***CHETOSTOMA*** Rondani, 1856

*Chetostoma stackelbergi* (Rohdendorf, 1955)

***CORNUTRYPETA*** Han, Wang & Kim, 1993

*Cornutrypeta spinifrons* (Schroeder, 1913)

*Cornutrypeta superciliata* (Frey, 1935)

***EULEIA*** Walker, 1835

= ***Cryptaciura*** Hendel, 1927

*Euleia heraclei* (Linnaeus, 1758)

*Euleia rotundiventris* (Fallén, 1814)

***MYOLEJA*** Rondani, 1856

*Myoleja lucida* (Fallén, 1826)

***PHILOPHYLLA*** Rondani, 1870

*Philophylla caesio* (Harris, 1776)

***RHAGOLETIS*** Loew, 1862

*Rhagoletis alternata* (Fallén, 1814)

*Rhagoletis cerasi* (Linnaeus, 1758)

*Rhagoletis meigenii* (Loew, 1844)

***TRYPETA*** Meigen, 1803

*Trypeta artemisiae* (Fabricius, 1794)

*Trypeta immaculata* (Macquart, 1835)

= *hamifera* Loew, 1846

*Trypeta zoe* Meigen, 1826

TEPHRITINAE Newman, 1834

Terellini Hendel, 1927

***CHAETORELLIA*** Hendel, 1927

*Chaetorellia jaceae* (Robineau-Desvoidy, 1830)

***CHAETOSTOMELLA*** Hendel, 1927

*Chaetostomella cylindrica* (Robineau-Desvoidy, 1830)

= *onotrophes* (Loew, 1848)

***ORELLIA*** Robineau-Desvoidy, 1830

*Orellia falcata* (Scopoli, 1763)

***TERELLIA*** Robineau-Desvoidy, 1830

**sg. *Cerajocera*** Rondani, 1856

*Terellia ceratocera* (Hendel, 1913)

*Terellia plagiata* (Dahlbom, 1850)

*Terellia tussilaginis* (Fabricius, 1775)

**sg. *Terellia*** Robineau-Desvoidy, 1830

*Terellia colon* (Meigen, 1826)

*Terellia ruficauda* (Fabricius, 1794)

*Terellia serratulae* (Linnaeus, 1758)

*Terellia winthemi* (Meigen, 1826)

Xyphosiini Hendel, 1927

***XYPHOSIA*** Robineau-Desvoidy, 1830

*Xyphosia miliaria* (Schrank, 1781)

Noeetini Norrbom & Korneyev, 1999

***ENSINA*** Robineau-Desvoidy, 1830

*Ensina sonchi* (Linnaeus, 1767)

***NOEETA*** Robineau-Desvoidy, 1830

= ***Oplocheta*** Rondani, 1856

*Noeeta pupillata* (Fallén, 1814)

Myopitini Bezzi, 1910

***EURASIMONA*** Korneyev & White, 1991

*Eurasimona stigma* (Loew, 1840)

***UROPHORA*** Robineau-Desvoidy, 1830

*Urophora aprica* (Fallén, 1820)

*Urophora cardui* (Linnaeus, 1758)

*Urophora cuspidata* (Meigen, 1826)

*Urophora jaceana* (Hering, 1935)

*Urophora solstitialis* (Linnaeus, 1758)

= *sonderupi* (Hering, 1940)

*Urophora stylata* (Fabricius, 1775)

Dithrycini Hendel, 1927

***DITHRYCA*** Rondani, 1856

*Dithryca guttularis* (Meigen, 1826)

Eutretini Munro, 1952

***XANTHOMYIA*** Phillips, 1923

= ***Paracarphotricha*** Hendel, 1927

*Xanthomyia alpestris* (Pokorny, 1887)

= *pseudoradiata* (Becker, 1900)

Tephritini Newman, 1834

***CAMPIGLOSSA*** Rondani, 1870

*Campiglossa absinthii* (Fabricius, 1805)

= *parvula* (Loew, 1862)

*Campiglossa argyrocephala* (Loew, 1844)

*Campiglossa difficilis* (Hendel, 1927)

= *tessellata* misid.

*Campiglossa grandinata* (Rondani, 1870)

= *borealis* Portschinsky, 1875

*Campiglossa guttella* (Rondani, 1870)

= *achyrophori* misid.

= *producta* misid.

*Campiglossa loewiana* (Hendel, 1927)

*Campiglossa plantaginis* (Haliday, 1833)

*Campiglossa punctella* (Fallén, 1814)

*Campiglossa solidaginis* (White, 1986)

*Campiglossa* sp. A

***DIOXYNA*** Frey, 1945

*Dioxyna bidentis* (Robineau-Desvoidy, 1830)

= *sororcula* auct. nec (Wiedemann, 1830)

***HERINGINA*** Aczél, 1940

*Heringina guttata* (Fallén, 1814)

***OXYNA*** Robineau-Desvoidy, 1830

*Oxyna flavipennis* (Loew, 1844)

*Oxyna nebulosa* (Wiedemann, 1817)

*Oxyna parietina* (Linnaeus, 1758)

***SPHENELLA*** Robineau-Desvoidy, 1830

*Sphenella marginata* (Fallén, 1814)

***TEPHRITIS*** Latreille, 1804

*Tephritis angustipennis* (Loew, 1844)

*Tephritis bardanae* (Schrank, 1803)

*Tephritis cometa* (Loew, 1840)

*Tephritis conura* (Loew, 1844)

*Tephritis dilacerata* (Loew, 1846)

*Tephritis fallax* (Loew, 1844)

*Tephritis hyoscyami* (Linnaeus, 1758)

*Tephritis leontodontis* (De Geer, 1776)

*Tephritis mutabilis* Merz, 1992

*Tephritis neesii* (Meigen, 1830)

= *nesii* misspelling

*Tephritis ruralis* (Loew, 1844)

Tephritis sp. cf. rydeni Hering, 1956

= *dioscurea* misid.

***TRUPANEA*** Schrank, 1795

*Trupanea stellata* (Fuessly, 1775)

Unplaced in Tephritinae (*incertae sedis*)

***ACINIA*** Robineau-Desvoidy, 1830

*Acinia corniculata* (Zetterstedt, 1819)

**ULIDIIDAE** Macquart, 1835

OTITINAE Aldrich, 1932

Myennidini Kameneva & Korneyev, 2006

***PSEUDOTEPHRITIS*** Johnson, 1802

*Pseudotephritis trypetoptera* (Hennig, 1939)

= *corticalis* auct. nec (Loew, 1873)

Otitini Aldrich, 1932

***CEROXYS*** Macquart, 1835

*Ceroxys urticae* (Linnaeus, 1758)

***HERINA*** Robineau-Desvoidy, 1830

*Herina frondescentiae* (Linnaeus, 1758)

*Herina paludum* (Fallén, 1820)

*Herina palustris* (Meigen, 1826)

***MELIERIA*** Robineau-Desvoidy, 1830

**sg. *Melieria*** Robineau-Desvoidy, 1830

*Melieria crassipennis* (Fabricius, 1794)

*Melieria omissa* (Meigen, 1826)

= *obscuripes* auct. nec (Loew, 1873)

***TETANOPS*** Fallén, 1820

**sg. *Eurycephalomyia*** Hendel, 1907

*Tetanops sintenisi* Becker, 1909

**sg. *Tetanops*** Fallén, 1820

*Tetanops myopinus* Fallén, 1820

ULIDIINAE Macquart, 1835

Seiopterini Kameneva & Korneyev, 1994

***HOMALOCEPHALA*** Zetterstedt, 1838

*Homalocephala albitarsis* Zetterstedt, 1838

= *bipunctata* (Loew, 1854)

*Homalocephala angustata* (Wahlberg, 1839)

*Homalocephala apicalis* (Wahlberg, 1839)

= *biseta* Frey, 1908

*Homalocephala bimaculata* (Wahlberg, 1839)

*Homalocephala biumbrata* (Wahlberg, 1838)

= *albitarsis* auct. nec Zetterstedt, 1838

***SEIOPTERA*** Kirby, 1817

*Seioptera vibrans* (Linnaeus, 1758)

Ulidiini Macquart, 1835

***PHYSIPHORA*** Fallén, 1810

*Physiphora alceae* (Preyssler, 1791)

= *demandata* (Fabricius, 1798)

## Checklist part 2: Sciomyzoidea

suborder Brachycera Macquart, 1834

clade Eremoneura Lameere, 1906

clade Cyclorrhapha Brauer, 1863

infraorder Schizophora Becher, 1882

clade Muscaria Enderlein, 1936

parvorder Acalyptratae Macquart, 1835

superfamily Sciomyzoidea Fallén, 1820

**COELOPIDAE** Hendel, 1910

***COELOPA*** Meigen, 1830

**sg. *Fucomyia*** Haliday, 1838

*Coelopa frigida* (Fabricius, 1805)

**DRYOMYZIDAE** Schiner, 1862

***DRYOMYZA*** Fallén, 1820

= ***Neuroctena*** Rondani, 1868

*Dryomyza anilis* Fallén, 1820

***DRYOPE*** Robineau-Desvoidy, 1830

*Dryope decrepita* (Zetterstedt, 1838)

*Dryope flaveola* (Fabricius, 1794)

***PARADRYOMYZA*** Ozerov, 1987

*Paradryomyza spinigera* Ozerov, 1987

***PSEUDONEUROCTENA*** Ozerov, 1987

*Pseudoneuroctena senilis* (Zetterstedt, 1838)

**HETEROCHEILIDAE** McAlpine, 1991

***HETEROCHEILA*** Rondani, 1857

*Heterocheila buccata* (Fallén, 1820)

**PHAEOMYIIDAE** Verbeke, 1950

***PELIDNOPTERA*** Rondani, 1856

*Pelidnoptera fuscipennis* (Meigen, 1830)

= *fumipennis* (Zetterstedt, 1846)

*Pelidnoptera nigripennis* (Fabricius, 1794)

**SCIOMYZIDAE** Fallén, 1820

SCIOMYZINAE Fallén, 1820

Sciomyzini Fallén, 1820

***COLOBAEA*** Zetterstedt, 1837

*Colobaea bifasciella* (Fallén, 1820)

*Colobaea distincta* (Meigen, 1830)

*Colobaea nigroaristata* Rozkošný, 1984

*Colobaea pectoralis* (Zetterstedt, 1847)

*Colobaea punctata* (Lundbeck, 1923)

***DITAENIELLA*** Sack, 1939

*Ditaeniella grisescens* (Meigen, 1830)

***PHERBELLIA*** Robineau-Desvoidy, 1830

*Pherbellia albocostata* (Fallén, 1820)

*Pherbellia alpina* (Frey, 1930)

*Pherbellia argyra* Verbeke, 1967

*Pherbellia brunnipes* (Meigen, 1838)

*Pherbellia cinerella* (Fallén, 1820)

*Pherbellia dubia* (Fallén, 1820)

*Pherbellia goberti* (Pandellé, 1902)

= *stylifera* Rozkošný, 1982

*Pherbellia griseicollis* (Becker, 1900)

= *lapponica* (Ringdahl, 1948)

*Pherbellia griseola* (Fallén, 1820)

*Pherbellia hackmani* Rozkošný, 1982

*Pherbellia nana* (Fallén, 1820)

*Pherbellia obscura* (Ringdahl, 1948)

*Pherbellia obtusa* (Fallén, 1820)

*Pherbellia pallidiventris* (Fallén, 1820)

*Pherbellia rozkosnyi* Verbeke, 1967

= *scutellaris* misid.

*Pherbellia schoenherri* (Fallén, 1826)

*Pherbellia sordida* (Hendel, 1902)

*Pherbellia stackelbergi* Elberg, 1965

*Pherbellia ventralis* (Fallén, 1820)

***PTEROMICRA*** Lioy, 1864

*Pteromicra angustipennis* (Staeger, 1845)

*Pteromicra glabricula* (Fallén, 1820)

*Pteromicra leucopeza* (Meigen, 1838)

*Pteromicra oldenbergi* (Hendel, 1902)

*Pteromicra pectorosa* (Hendel, 1902)

***SCIOMYZA*** Fallén, 1820

*Sciomyza dryomyzina* Zetterstedt, 1846

*Sciomyza sebezhica* Przhiboro, 2001

*Sciomyza simplex* Fallén, 1820

***TETANURA*** Fallén, 1820

*Tetanura pallidiventris* Fallén, 1820

Tetanocerini Newman, 1834

***ANTICHETA*** Haliday, 1839

*Anticheta analis* (Meigen, 1830)

*Anticheta atriseta* (Loew, 1849)

*Anticheta brevipennis* (Zetterstedt, 1846)

*Anticheta nigra* Karl, 1921

= *nigroaenea* Frey, 1935

***COREMACERA*** Rondani, 1856

*Coremacera marginata* (Fabricius, 1775)

= *tristis* (Harris, 1780) preocc.

***DICHETOPHORA*** Rondani, 1868

*Dichetophora finlandica* Verbeke, 1964

***DICTYA*** Meigen, 1803

*Dictya umbrarum* (Linnaeus, 1758)

***ECTINOCERA*** Zetterstedt, 1838

*Ectinocera borealis* Zetterstedt, 1838

***ELGIVA*** Meigen, 1838

*Elgiva cucularia* (Linnaeus, 1767)

*Elgiva divisa* (Loew, 1845)

*Elgiva solicita* (Harris, 1780)

= *sundewalli* Kloet & Hincks, 1945

***EUTHYCERA*** Latreille, 1829

*Euthycera chaerophylli* (Fabricius, 1798)

*Euthycera fumigata* (Scopoli, 1763)

***HYDROMYA*** Robineau-Desvoidy, 1830

*Hydromya dorsalis* (Fabricius, 1775)

***ILIONE*** Haliday in Curtis, 1837

= ***Tumidicerus*** Knutson & Berg, 1967

**sg. *Ilione*** Haliday in Curtis, 1837

*Ilione lineata* (Fallén, 1820)

**sg. *Knutsonia*** Verbeke, 1964

*Ilione albiseta* (Scopoli, 1763)

***LIMNIA*** Robineau-Desvoidy, 1830

*Limnia paludicola* Elberg, 1965

*Limnia unguicornis* (Scopoli, 1763)

***PHERBINA*** Robineau-Desvoidy, 1830

*Pherbina coryleti* (Scopoli, 1763)

***PSACADINA*** Enderlein, 1939

*Psacadina zernyi* (Mayer, 1953)

= *punctata* misid.

***RENOCERA*** Hendel, 1900

*Renocera pallida* (Fallén, 1820)

*Renocera striata* (Meigen, 1830)

*Renocera stroblii* Hendel, 1900

= *fuscinervis* auct. nec (Zetterstedt, 1838)

***SEPEDON*** Latreille, 1804

**sg. *Sepedon*** Latreille, 1804

*Sepedon sphegea* (Fabricius, 1775)

*Sepedon spinipes* (Scopoli, 1763)

***TETANOCERA*** Duméril, 1800

**sg. *Chaetotetanocera*** Mayer, 1953

*Tetanocera robusta* Loew, 1847

**sg. *Tetanocera*** Duméril, 1800

? *Tetanocera amurensis* Hendel, 1909

*Tetanocera arrogans* Meigen, 1830

*Tetanocera elata* (Fabricius, 1781)

*Tetanocera ferruginea* Fallén, 1820

= *brunnipennis* Frey, 1924

*Tetanocera freyi* Stackelberg, 1963

*Tetanocera fuscinervis* (Zetterstedt, 1838)

= *unicolor* Loew, 1847

*Tetanocera hyalipennis* von Roser, 1840

*Tetanocera kerteszi* Hendel, 1901

= *griseicollis* Frey, 1924

= *ornatifrons* Frey, 1924

*Tetanocera lapponica* Frey, 1924

*Tetanocera latifibula* Frey, 1924

*Tetanocera montana* Day, 1881

= *borealis* Frey, 1924

*Tetanocera phyllophora* Melander, 1920

= *nigricosta* misid.

*Tetanocera silvatica* Meigen, 1830

***TRYPETOPTERA*** Hendel, 1900

*Trypetoptera punctulata* (Scopoli, 1763)

**SEPSIDAE** Walker, 1833

ORYGMATINAE Frey, 1921

***ORTALISCHEMA*** Frey, 1925

*Ortalischema albitarse* (Zetterstedt, 1847)

SEPSINAE Walker, 1833

***THEMIRA*** Robineau-Desvoidy, 1830

*Themira annulipes* (Meigen, 1826)

*Themira arctica* (Becker, 1915)

*Themira biloba* Andersson, 1975

*Themira germanica* Duda, 1926

*Themira gracilis* (Zetterstedt, 1847)

*Themira leachi* (Meigen, 1826)

*Themira lucida* (Staeger, 1844)

*Themira malformans* Melander & Spuler, 1917

*Themira minor* (Haliday, 1833)

*Themira nigricornis* (Meigen, 1826)

*Themira paludosa* Elberg, 1963

*Themira pusilla* (Zetterstedt, 1847)

*Themira putris* (Linnaeus, 1758)

*Themira superba* (Haliday, 1833)

***SALTELLA*** Robineau-Desvoidy, 1830

*Saltella sphondylii* (Schrank, 1803)

***NEMOPODA*** Robineau-Desvoidy, 1830

*Nemopoda nitidula* (Fallén, 1820)

= *cylindrica* (Fabricius, 1794) preocc.

*Nemopoda pectinulata* Loew, 1873

*Nemopoda speiseri* (Duda, 1926)

***MEROPLIUS*** Rondani, 1874

*Meroplius fukuharai* (Iwasa, 1984)

*Meroplius minutus* (Wiedemann, 1830)

= *stercorarius* (Robineau-Desvoidy, 1830)

***SEPSIS*** Fallén, 1810

*Sepsis biflexuosa* Strobl, 1893

*Sepsis cynipsea* (Linnaeus, 1758)

*Sepsis duplicata* Haliday, 1838

= *pilipes* van der Wulp, 1871

*Sepsis flavimana* Meigen, 1826

= *borealis* Frey, 1825

*Sepsis fulgens* Meigen, 1826

= *communis* Frey, 1925

*Sepsis luteipes* Melander & Spuler, 1917

= *lamellifera* Frey, 1925

*Sepsis nigripes* Meigen, 1826

*Sepsis orthocnemis* Frey, 1908

*Sepsis punctum* (Fabricius, 1794)

= *luteipes* misid.

*Sepsis thoracica* (Robineau-Desvoidy, 1830)

*Sepsis violacea* Meigen, 1826

## Excluded species

*Allopiophila dudai* (Frey, 1930) not found within present borders

*Bactrocera* sp. imported with fruit

*Campiglossa irrorata* (Fallén, 1814) not found within present borders

*Ceratitis capitata* (Wiedemann, 1824) imported with fruit

*Earomyia crystallophila* (Becker, 1895) not found within present borders

= *nigroviolacea* (Frey in Lundström & Frey, 1913)

*Lonchaea hirticeps* Zetterstedt, 1838 misidentified

*Orygma luctuosum* Meigen, 1830 not found within present borders

*Pherbellia dorsata* (Zetterstedt, 1846) misidentified

*Tephritis nigricauda* (Loew, 1856) misidentified

## Notes

***Allopiophila dudai* (Frey, 1930)**. This species was described by Frey (1930) from the Kola peninsula (Murmansk Oblast, Russia). Frey et al. (1941) and Hackman (1980) listed it from Finland, but to our knowledge it has never been reliably recorded from this country.

***Allopiophila* sp. A**. An apparently undescribed arctic species near *Allopiophila vulgaris*.

***Campiglossa* sp. A** is a distinct, possibly undescribed species of *Campiglossa*. It is common on *Saussurea alpina* in Northern Finland (Juhani Itämies, priv. comm.).

***Lonchaea bruggeri* Morge, 1967**, ***Lonchaea contigua* Collin, 1953** and ***Lonchaea difficilis* Hackman, 1956**. No males have been confirmed from the country. See Winqvist and Kahanpää (2007) for more details.

***Lonchaea bukowskii* Czerny, 1934**. No males have been confirmed from the country.

**Tephritis sp. cf. rydeni Hering, 1956**. Finnish material formerly assigned to *Tephritis dioscurea* (Loew, 1856) belongs to a currently unrecognised species that may be *Tephritis rydeni* Hering (Söderman et al. 2007, Winqvist and Kahanpää 2007).

***Tetanocera amurensis* Hendel, 1909**. Recorded from Finland in Fauna Europaea (Rozkošný and Knutson 2011) and known from Estonia and Northwestern European Russia (Elberg 1988). We have been unable to find any Finnish material or primary published records.
